# The Hospital World

**Published:** 1910-10-01

**Authors:** 


### THE HOSPITAL WORLD.

### Elections and Appointments.

MISS MARY WARDELL has been appointed a nursing sister in Queen Alexandra's Military Nursing Service for India.

MR. HERBERT MONCKTON, formerly Town Clerk of Maidstone, and brother of the late Sir John Monckton, Town Clerk of the City of London, has been appointed Chairman of the Kent County Ophthalmic Hospital.

THE following admission committee has been appointed at the Royal Victoria Hospital, Belfast, for the months of October, November, and December: Mr. William Armstrong, chairman; Mr. H. Berrington, and Mr. W. Carson.

### Personalia.

THE new director of the Eppendorf Hospital, in which the chief staff office was rendered vacant through the recent death of Professor Lenhartz, is Professor Brauer, of Marburg. Professor Brauer is comparatively unknown in this country as a hospital expert, but in Germany he is reputed a highly qualified hygienist who has had considerable institutional experience. It is stated that several novel methods will shortly be introduced at Eppendorf.

MR. L. K. KNIGHT, who for seven years has acted as hon. secretary to the Committee of the Hove hospital parade, had been nominated as its representative on the Board of the Brighton, Hove, and Preston Dispensary. This appreciation of his services is timely, for anyone who has had experience in organising a parade, and in keeping alive the public's interest in it as an annual event not grown stale, knows the anxious care and the funds of energy which are exacted by this work. Mr. Knight's experience will make him a valuable addition to the board.

THE death took place on Saturday last, at Beer, Devon, of Captain John Gaspard Le Marchant, late 7th Hussars, the eldest son of the late General Sir John Gaspard Le Marchant, Lieutenant-Governor of Newfoundland, Halifax, and Malta, and Commander-in-Chief at Madras. Captain Le Marchant served in India in 1861-62 with his regiment, and was mentioned in despatches for his services. After his retirement from the Army he devoted himself to charitable work in London, and was for many years a secretary of the Charity Organisation Society, to which he devoted much time and interest. He was also for some time chairman of the Board of Guardians of St. George's, Hanover Square, secretary to the Officers' Families Association, and of the Newport Market Refuge for Boys, and interested himself in many other philanthropic and institutional works.

THE Kent County Ophthalmic Hospital has lost the active services of an old and tried friend by the retirement of Sir Charles Whitehead from the chairmanship. It is often our privilege in this column to record long spells of work honourably completed, but it is not often that we are able to congratulate a hospital worker on so long a service in one office as Sir Charles has completed just now. For thirty-five years he has been Chairman, and in that time must have marked the astonishing development of his office, side by side with the growth of the hospitals, and of the interest of the public in their advance. We believe a glance through the minute books would reveal that in other ways, besides those open to the Chairman, Sir Charles Whitehead has done good work for his institution, and we congratulate him on having borne so long the responsibilities of this important post.

DR. GEORGE C. PEACHEY, who has just finished the third part of his history of St. George's Hospital, has lately been an inmate of his old hospital for an attack of appendicitis, which necessitated operation. His many friends will be gratified to hear that he is making good progress. The first part of his "History," which promises to be the best historical and critical work on any London hospital, was published some months ago, while the second was issued only recently, and deals with the conversion of Lanesborough House into the modern hospital of St. George. We hope to publish an extended review of the work in an early number of THE HOSPITAL.

THE death took place last week, at his residence, Hartsdown, Margate, of Captain Charles Taddy Hatfeild, late King's Dragoon Guards, a well-known figure in the local life of the citizens. Since 1874, when he retired from the service on the death of his father, Captain Hatfeild took an active interest in the affairs of his neighbourhood. He was made a J.P. and D.L. for Kent, and was a vice-president of Margate Cottage Hospital, which gained considerably from having among its official supporters a man who came into contact with most interests in the county. As Cinque Port magistrate and president of the Margate Lifeboat Institution, to juxtapose two of his diverse interests, he was near the focus of county interests, and led the life of a patriotic citizen, to whom no form of local public life was unimportant or uninteresting. It is this type of man, not perhaps too common, that makes the office of hospital vice-president a useful adjunct to an institution by connecting it officially with public opinion on many sides.

LOUISA LADY DE ROTHSCHILD'S death at Aston-Clinton will be felt in all charitable circles, with which her advanced age had identified her for so long. A Jewish correspondent in the *Times* brings out the character of her philanthropy thus forcibly: "Her philanthropy did not consist merely in signing cheques and acting as figurehead to charitable institutions; she took a personal interest in every one of the many acts of benevolence with which she was associated. . . . She was especially interested in the eyesight of the children, and for many years paid the cost of spectacles for those children who needed them at two large East-end schools. She warmly supported the movement for country holidays for poor children, and the parties of children who were sent under the auspices of the Children's Country Holidays Fund to the Tring centre were invariably entertained at her expense." No apter instance will be wanted of the clear-sighted benevolence of Lady de Rothschild than this of the free gift of spectacles. It showed that she appreciated the increasing importance in which the children would be regarded by the statesmen of to-day. Her action is but another example of the way in which private philanthropy finds its function in pioneer work, which is followed in time by communal action. The late Mr. Kirkman Gray would have rejoiced rightly in this illustration of his pet thesis.

